# A Sponge-Like Double-Layer Wound Dressing with Chitosan and Decellularized Bovine Amniotic Membrane for Promoting Diabetic Wound Healing

**DOI:** 10.3390/polym12030535

**Published:** 2020-03-02

**Authors:** Yang Yang, Yanyan Zhang, Yishu Yan, Qian Ji, Yutong Dai, Suyuan Jin, Yanxian Liu, Jinghua Chen, Liping Teng

**Affiliations:** 1 Key Laboratory of Carbohydrate Chemistry and Biotechnology, Ministry of Education, School of Pharmaceutical Sciences, Jiangnan University, Wuxi 214122, China; yangy@jiangnan.edu.cn (Y.Y.); yanyanzhang223@163.com (Y.Z.); yanyishu@jiangnan.edu.cn (Y.Y.); jiqian@jiangnan.edu.cn (Q.J.); d853304174@sina.com (Y.D.); a17851311170@163.com (S.J.); liuyanxian1998@gmail.com (Y.L.); 2 School of Medicine, Jiangnan University, Wuxi 214122, China

**Keywords:** chitosan, decellularized bovine amniotic membrane, diabetic wound healing, double-layer dressing

## Abstract

The diabetic wounds do not heal easily in part because they are susceptible to infection due to environmental influences. Wound dressing is crucial to wound healing, as it can basically protect the wound from external damages and provide a suitable microenvironment for tissue regeneration. In this study, a double-layer membrane that consists of chitosan sponge and decellularized bovine amniotic membrane (dBAM) has been developed by freeze-casting method. The results showed that the porous structure of the sponge layer improved the performances of blood coagulation and swelling. The dense dBAM can optimize the mechanical property of wound dressing. In vitro studies revealed that the bilayer membrane had favorable biocompatible, especially for human foreskin fibroblast cells (HFF-1) cell adhesion and proliferation. Moreover, the full-thickness skin defects of diabetic model mice that treated with bilayer membrane showed over 80% closure in 8 days. Our findings imply that the double-layer dressing has great potentials to be used in diabetic patients.

## 1. Introduction

As the largest human organ, skin is responsible for not only the physical protection but also for sensory detection, thermoregulation, immune surveillance, etc. [1]. In general, skin can be recovered to minimal scar through a complex interaction process. However, it is quite hard to repair once the wound has become chronic, such as diabetic foot ulcers [2]. Many diabetic patients suffer from the complicate chronic refractory wound [3]. The pathogenesis of diabetic foot ulcers is not well understood, but some work has exhibited that growth factors and immune suppressive cytokine can improve wound healing [4]. Furthermore, the wound dressing is also reported to be vital to healing process, mainly because it can protect the wound from external damages as well as provides a suitable microenvironment for tissue regeneration.

Recently, quite a few types of wound dressing materials have been designed and applied to the chronic-wounds, such as hydrogel [5,6,7], nanofiber [8,9,10], sponge [11,12,13], etc. Suitable dressings should accord with the characteristics of excellent biocompatibility, high elasticity and air permeability in order to effectively improve wound healing. In addition, the dressing must also have excellent physical swelling and antibacterial property, since the chronic wound surface is easy to suffer from exudate infiltration and serious bacterial infection [14]. In comparison, porous sponges with superior blood coagulation, adsorption performance, and shape recovery properties have been investigated as a potential wound dressing. For instance, Cheng has developed a polysaccharide-based sponges and proved that hybrid sponges were non-toxic, with a great performance in promoting blood coagulation [3]. The sponges can also be modified by the addition of antibacterial agents [12] or growth factors [15] to improve the therapeutic effects. Chitosan (CS), the biocompatible and antimicrobial biomaterial, is generally selected to prepare the sponge for wound healing [16,17,18,19]. Moreover, it is reported that CS can influence the fibroblast activation, cytokine production, giant cell migration, and stimulation of type IV collagen synthesis during the wound healing process [20,21]. Part of sponge as wound dressing is so fragile that debris tends to remain in the wound, resulting in the secondary injury to the wound when removing sponges. Thus, improving the flexibility of sponges will be helpful to increase their potential for use as wound dressings.

Since the early 20th century, animal amniotic membrane (AM) has been widely applied for disease treatments such as severe eye injury, diabetic neuron vascular ulcers and wound healing [22,23,24,25]. Amniotic membrane is composed of the basement membrane and stroma, which is covered by a layer of epithelial cells. Amniotic membrane is rich in collagen, growth factors, and immune suppressive cytokine such as interleukin-4 and interleukin-10 [26,27,28]. However, human AM is insufficient to provide a mass production and the use of human AM has led to religious concerns. Studies showed that fresh or decellularized bovine AM (dBAM) provided similar efficacy for wound healing as the fresh human AM [29,30,31]. Therefore, the use of bovine AM (BAM) as an alternative might address the issue of above limitations. In recent years, acellular tissue scaffolds have attracted great interests because of their low immunogenicity, high biocompatibility, good biodegradability, and high similarity in the architecture and composition of extracellular matrix (ECM) to the target tissue [32]. Moreover, some researches indicated that the good mechanical properties of amniotic membrane made it suitable for ocular surface repair and its dynamic elastic shear modulus could reach more than 400 Pa at 1 rad/s [33,34,35]. Therefore, the dBAM is more appropriate as a biological dressing.

Considering that dBAM can provide rich collagen and growth factors to accelerate the wound healing along with the chitosan sponge can improve the absorption capacity for fluid, fill the deep wound, and accelerate the blood coagulation, herein, a dBAM combined sponge-like CS membrane (BAMCSM) as a wound dressing was fabricated by freeze-casting method. BAMCSM consisted of two layers of chitosan sponge and dBAM, which was crosslinked by poly (ethylene glycol) diglycidyl ether (PEGDGE). Using chitosan as a precursor and poly (ethylene glycol) diglycidyl ether (PEGDGE) as a cross-linking agent, the membrane could be simultaneously cross-linked via a ring-opening polymerization reaction, while the amino group of chitosan and dBAM could react with the epoxy group of PEGEGE. This method aimed to maximize the retention of biomedical and architectural properties of dBAM within a composite membrane. The BAMCSM was characterized by several methods, including the composition analysis of dBAM, scanning electron microscopy (SEM), mechanical testing, swelling behavior, in vitro blood coagulation test. In addition, we tested the effects of BAMCSM on the human foreskin fibroblast cells (HFF-1) in vitro and on diabetic wound healing in mice in vivo.

## 2. Materials and Methods

### 2.1. Materials

Chitosan (CS, the viscosity is 100–200 mPa s, the deacetylation degree ≥95%) and poly (ethylene glycol) diglycidyl ether (PEGDGE, Mn = 500, purity ≥95%) were purchased from Shanghai Aladdin Biochemical Technology Co., Ltd. (Shanghai, China). Trypsin (3500 U/mg) was purchased from Shanghai Macklin Biochemical Co., Ltd. (Shanghai, China). Dodecyl sodium sulfate, Agar-agar powder, Sodium chloride (≥99.5%), Disodium hydrogen phosphate dodecahydrate (≥99.0%), Potassium dihydrogen phosphate (≥99.5%) and Potassium chloride (≥99.5%) was purchased from Sinopharm Chemical Reagent Co., Ltd. (Shanghai, China). Human foreskin fibroblasts (HFF-1) were provided by Shanghai cell bank of Chinese academy of sciences. Dulbecco’s minimum essential medium (DMEM) and fetal bovine serum (FBS) were purchased from Sciencecell (Santiago, CA, USA).

### 2.2. BAM Decellularization

To remove cellular components completely, four decellularized protocols were comparatively evaluated. Fresh BAMs were obtained from bovine placentas which purchased form the market. After washing with phosphate-buffered saline solution, the BAMs were transferred into 0.25% and 0.5% trypsin solutions with gentle shaking for 2 h at room temperature. Then, the BAMs were disposed by 1% SDS solutions after different durations (12 h and 24 h) at room temperature. After washing in deionized water three times, the membranes were dried naturally and kept at 4 °C for the subsequent analyses. According to the result of staining with DAPI, the best condition was chosen after analysis of the DNA residues. In addition, the component contents of dBAM have been characterized, including water, total protein, polysaccharides, fat, etc.

### 2.3. dBAM Modification with Chitosan

**Figure 1:**
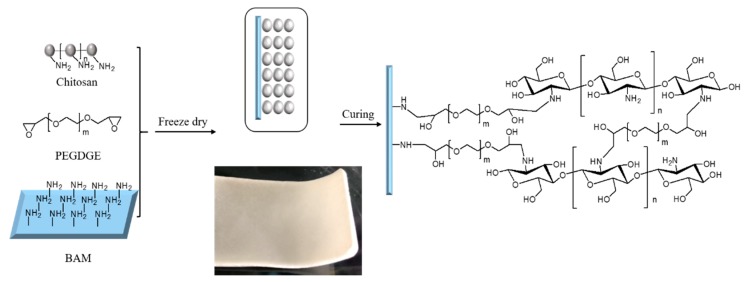
Schematic illustration of the preparation of double-layer membrane.

The preparation process of BAMCSM is shown in Figure 1. All membranes were prepared according to the following steps. The dry dBAM was cut into rectangular shape (10 cm long and 3 cm wide) and placed into a polytetrafluoroethylene mold. A certain amount of chitosan was added to 1% acetic acid solution and sonicated for 30 min until a homogeneous solution was obtained with the concentration of 20 mg/mL. The cross-linking reagent (PEGDGE) was added into the solution of chitosan with a mass ratio of 1/5 and sonicated for 10 min. The mixed solution was centrifuged at 4000 rpm for 20 min to remove air bubbles and then transferred to the plate over dBAM. The mass ratio of the resulting solution and dBAM was 1/1 and 1/3 respectively which are named M1-1 and M1-3. The plate of the mold was put into frozen at −80 °C for 12 h followed by lyophilizing for 24 h to remove the solvent. Then, the mold was placed at 40 °C for 6 h to ensure sufficient curing of chitosan, dBAM and the cross-linking reagent. The obtained membrane was washed by deionized water for several times and vacuum-dried. The membrane was stored at 4 °C.

### 2.4. Morphological Analysis and Mechanical Testing

Both chitosan, dBAM, and M1-1 were subjected to attenuated total reflectance infrared measurements with scanning ranger 400–4000 nm using a BRUKER FI-IR (Bruker, Karlsruhe, Germany). The data was processed by the software (Bruker OPUS 6.5) with smoothing and normalization. Afterwards, the images were obtained using Origin.

The pore morphologies of the two-layer membranes were observed with scanning electron microscope (MERLIN Compact, Zeiss, Oberkochen, Germany). ALL membranes were fractured after quenching in liquid nitrogen to obtain an interior cross-section and then were sputter-coated with a thin layer of gold for the investigation of internal topographies.

The mechanical properties of membranes were measured by tensile machine (MST, Model E43) to compare the tensile stress between a single-layer chitosan membrane and the double-layer membranes. The rectangular membranes (the rectangle of 10 cm long and 3 cm wide) containing a monolayer of chitosan were prepared, named M1-1 and M1-3. All measurements were repeated three times for each kind of membrane.

### 2.5. Swelling Ability

The swelling ability of dBAM, M1-1, and M1-3 was evaluated after putting it into PBS solutions for 6 h at room temperature, respectively. The excess water of the samples was removed gently after predetermined period. Then the samples were weighed immediately and the swelling ratio was calculated using the following equation.

$$\mathrm{Swelling}\;\left(\%\right)=\frac{W_{t}-W_{0}}{W_{0}}\times 100$$

where W_t_ and W_0_ are the wet and dry weight of dBAM, M1-1, and M1-3, respectively.

### 2.6. In Vitro Blood Coagulation Test

The medical gauze (control), dBAM, M1-1, and M1-3 were cut into circular shapes (diameter is about 15 mm) and placed on culture dishes. Next, 100 μL of blood which containing 10% sodium citrate was dropped onto the surface respectively. The membranes containing blood were incubated at 37 °C for 5 min. Afterwards, 50 mL of distilled water was added to the culture dish from the edge softly. The dissociated red blood cells were hemolyzed in water. Using the hemoglobin test solution (C021, Nanjing Jiangsu, China) to quantify the blood clots on each material (gauze, dBAM, M1-1 and M1-3). The absorbance of each sample was measured by an UV-visible spectrophotometer at a wavelength of 540 nm. Each group was repeated six times. Hemoglobin content of samples was measured by the following Equation [13].

$$\mathrm{Hemoglobin}\;\mathrm{content}\;\left(g/L\right)=(OD-OD_{0})\times 367.7$$

where *OD*_0_ means the blank absorbance value and *OD* means the absorbance value of the sample.

### 2.7. Biocompatibility Testing (Cell Viability Assay)

The cytotoxic effect of the produced membranes on HFF-1 was evaluated in vitro following ISO 10993-5 and ISO 10993-12. The membranes were first sterilized by UV irradiation over 30 min, and then immersed in the cell medium for 24 h (the extraction ratio of dBAM and M1-1 is 6 cm^2^/mL and M1-3 is 3 cm^2^/mL). Therefore, the membranes were removed and the extraction medium was referred to conditioned medium. The HFF-1 was incubated with the conditioned medium in humidified incubator for 24 h. The control group was incubated with the normal cell medium. Cell viability was measured using the MTT method at specified time points. Briefly, 0.5 mg/mL MTT solution (100 μL) was added to the cell culture plates for staining living cells for 4 h. Afterwards, the MTT was removed and 100 μL DMSO was added to the culture plates, incubating for 15 min. Subsequently, the results were tested at 570 nm with Microplate reader (Bio-Rad 680).

### 2.8. Animal Studies

This study was approved by the Ethical Approval for Research Involving Animals of Jiangnan University (NO. JN. No 20190530c0500915). The male C57BL/6 mice weighing 20–25 g were chosen to evaluate the wound healing behavior by covering the dressing materials. The animals were kept at room temperature in a 12 h light/dark cycle and had free access to pellet diet and water. All the experiments were operated in a sterile environment.

Diabetic model was induced by continuous intraperitoneal injection of the streptozotocin (70 mg/kg) solution, which was dissolved in a citrate buffer at pH 4. One week after the injection, the fasting blood-glucose level of mice was above 13.5 mmol/L was believed to be diabetic.

The mice were randomly divided into four groups including control (Tegaderm Film, 3M dressing, USA), drug (Yunnan Baiyao, China), M1-1, and M1-3. The mice were anesthetized by intraperitoneal injection of avertin. Then they were depilated the back hair and operated a full-thickness skin wound with approximately 10 mm diameter. The wound sizes were measured on the 3rd, 5th, 8th, and 14th day. The wound healing ratio (HR) was calculated by the Equation as follows.

$$\mathrm{HR}\left(\%\right)=\frac{S_{0}-S_{X}}{S_{0}}\times 100$$

where S_0_ and S_X_ are the wound areas at the beginning day and the predetermined experimental day, respectively.

### 2.9. Histomorphological Assessment of Wound Healing

For the histomorphology assessment, the wound tissues were evaluated on day 3, 5, and 14 which fixed in 4% paraformaldehyde and embedded in paraffin. The wound sections were subjected to histomorphological analysis by H&E and Masson staining. In addition, the thickness of granulation tissue was measured through histomorphological test.

## 3. Results

### 3.1. Characterization of the dBAM and BAMCSM

Four decellularization protocols were used to remove the cellular components. The result of DAPI staining confirmed that the condition of 0.5% trypsin solutions with gentle shaking for 2 h and 1% SDS solutions with 24 h was optimal and adopted for the further experiments (Figure S1). With this condition, there were no intact nucleuses or DNA left on the dBAM. In addition, the components of BAM have been measured and the results have been shown in Table S1. The contents of water, total protein (including collagen) polysaccharide and fat were 11.8%, 66.62%, 22.25%, and 0.12%, respectively. The content of collagen was 21.77%, measured by hydroxyproline method. Table S2 showed the theoretical weight of each membranes (rectangular shape of 10 cm long and 3 cm wide).

In this research, the cross-linking reagent was PEGDGE which could copolymerize with chitosan. ATR-FTIR was employed to analyze PEGDGE, chitosan, and M1-1. As shown in Figure S2, the basic characteristic peaks were consistent with both chitosan and M1-1. The peak at 3332 cm^−1^ corresponding to the overlapped stretching vibrations between -OH and -NH groups. The peaks at 2918 and 2851 cm^−1^ could be attributed to the-CH stretch. The peaks at 1648, 1540, and 1074 cm^−1^ could be attributed to amide II band due to C-O, N-H stretching and skeletal vibrations involving C-O stretch, respectively. The above peaks existed both in chitosan and M1-1. In the reaction, the amino group of chitosan and dBAM would react with the epoxy group of PEGEGE. The peak of epoxy group of PEGDGE could be found at 910 cm^−1^. The disappearance of the peak at 910 cm^−1^ in M1-1 indicated that the epoxy group of PEGDGE was copolymerized with the amino group of chitosan successfully.

**Figure 2:**
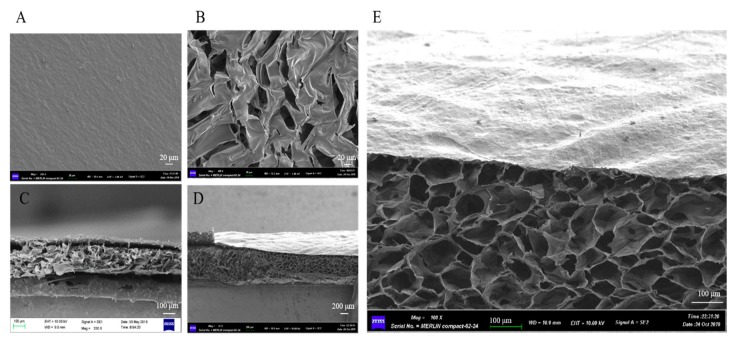
The morphology of double-layer membrane, (**A**) the surface of decellularized bovine amniotic membrane (dBAM), (**B**) the surface of sponge, (**C**) the cross profile of M1-1, (**D**) the cross profile of M1-3, (**E**) the enlarged profile of M1-3.

In order to observe the morphology, the dBAM, M1-1, and M1-3 were characterized by SEM. Figure 2 displayed the cross-section morphology of dBAM and BAMCSM (M1-1 and M1-3), respectively. According to Figure 2A,B, it was obvious that the two layers have different characters with a dense layer of dBAM and a polyporous layer of chitosan. Figure 2C,D showed the cross profiles of M1-1 and M1-3. The two layers were cross-linked together and M1-3 was almost three times as thick as M1-1. Figure 2E was the enlarged profile of M1-3 which showed a regular polyporous structure. The results suggested that the new dressing had characteristics of soft, good absorbability, and breathability. In conclusion, this material had the potential to be a good dressing.

### 3.2. Membrane Swelling Ability and Mechanical Testing

**Figure 3:**
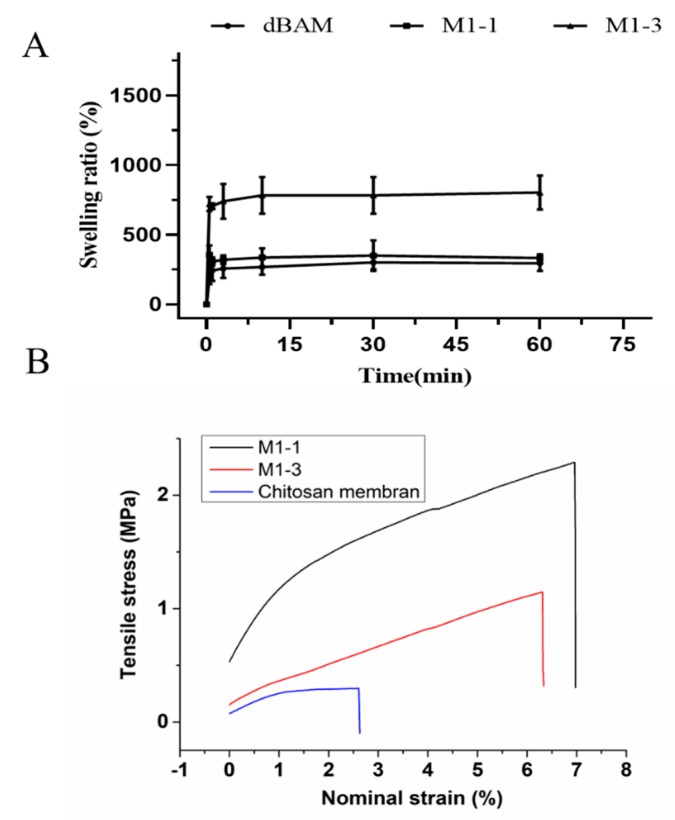
(**A**) Swelling ratios of dBAM, M1-1, and M1-3 (0–60 min), (**B**) the mechanical testing of chitosan membrane, M1-1, and M1-3.

The swelling behavior of membranes (dBAM, M1-1, and M1-3) was evaluated in PBS buffer during 6 h. As shown in Figure 3A, the membranes absorbed the PBS rapidly in a minute and the swelling ratios were determined to be approximately 300%, 400%, and 800%, respectively. Swelling test was evaluated in PBS for 6 h as shown in Figure S3. More specifically, the Figure 3A showed the points before 60 min. By comparing the swelling ratios, the improvement of swelling performance could be attributed to the sponge-like structure of chitosan. It indicated that two-layer membrane could absorb the wound fluid rapidly providing a better environment for wound healing.

The main disadvantage of the sponge structure is its poor mechanical property which is unfavorable to debridement and the wound healing. Figure 3B displayed a better mechanical property of the double-layer dressing than that of monolayer chitosan sponger obviously. Therefore, the dBAM layer could maintain the membrane structure and benefit the wound cleaning. The stress at the breakage of the monolayer chitosan, M1-1, and M1-3 were 0.29 MPa, 2.29 MPa, 1.15 MPa, respectively, and the maximum nominal strain were 2.60%, 6.97%, 6.31%, respectively. The result suggested that the combination of dBAM and the sponge-like chitosan improved the performance of wound dressing.

### 3.3. In Vitro Blood Coagulation Test

**Figure 4:**
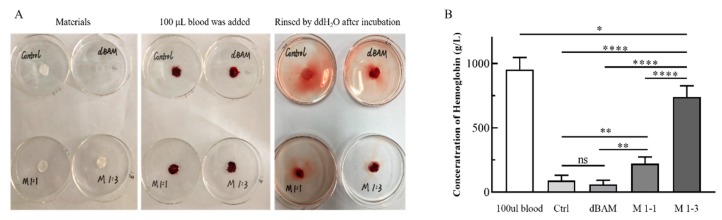
In vitro blood coagulation test, (**A**) the photographs of the blood-clotting process, (**B**) the concentration of hemoglobin in the blood clots on the materials (ns: *p* > 0.05; *: *p* < 0.05; **: *p* < 0.01; ****: *p* < 0.0001).

The hemostatic ability of materials was evaluated by the blood coagulation test. According to Figure 4A, the water of rinsing control (medical gauze) and dBAM turned red, which mean that the medical gauze and dBAM were difficult for blood coagulation. The rinsing water of M1-1 was slightly red and the M-3 was almost transparent. A lighter color of the rinsing water means faster clotting. The phenomenon indicated that the thickness of the sponge-like layer was crucial to the blood coagulation speed. In addition, the hemoglobin concentrations on the different membranes were quantitatively analyzed by UV detector and the concentration of hemoglobin in 100 μL blood was set as the reference. The average concentrations of the 100 μL blood, medical gauze, M1-1 and M1-3 were 919.25, 113.99, 72.80, 292.42, and 685.39 g/L, respectively. These results suggested that the sponge-like structure greatly increased the hemostatic ability of wound dressing. In addition, the positive charge of the amino groups of CS could be combined with the negative charge of the platelets to accelerate hemostasis [36].

### 3.4. Biocompatibility of dBAM and BAMCSM

**Figure 5:**
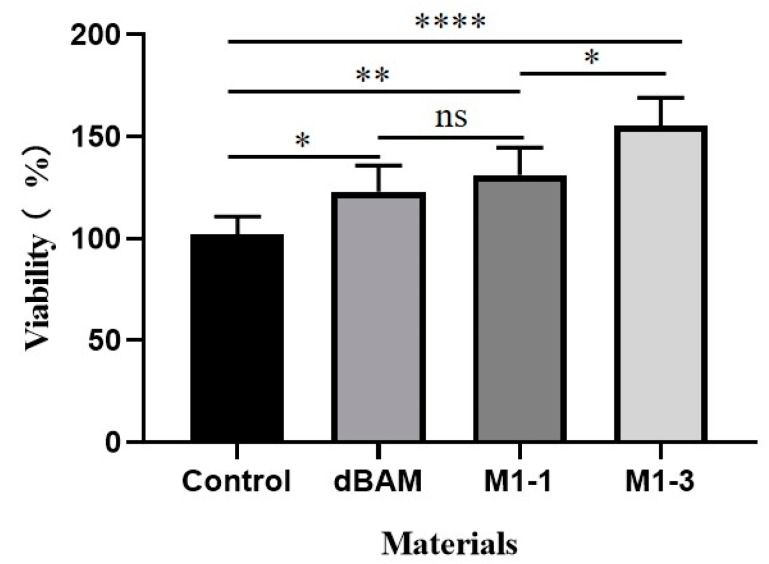
The biocompatibility test of dBAM, M1-1, and M1-3 (*: *p* < 0.05; **: *p* < 0.01; ****: *p* < 0.0001).

HFF-1 cell plays a major role in the wound healing which was chosen to evaluate the biocompatibility of dBAM and BAMCSM. At first, the HFF-1 was incubated with the conditioned medium in humidified incubator for 24 h. Then, MTT method was used to examine the cell viability and the result was shown in Figure 5. The cell viability of dressing groups was more than 100% which indicated that the dressing groups showed a higher proliferation rate of cells than control group. Accordingly, the HFF-1 showed great viability, indicating that all membranes had excellent biocompatibilities. According to literatures [29,31], dBAM is rich in collagen and has numerous growth factors which can promote the wound healing. As shown in Figure 5, the cell of viability of dBAM group is higher than control group. On the other hand, chitosan is reported as an accelerator of wound healing which with higher degrees of deacetylation and lower molecular weight strongly stimulate fibroblast proliferation [37]. The mechanism research of chitosan accelerates the fibroblast proliferation suggests that, chitosan effectively promotes wound repair process by accelerating the proliferation of fibroblasts as its ability of promoting the production of TGF-β1 and platelet-derived growth factor as well as stimulating IL-8 in fibroblasts to regulate the collagen secretion [38,39]. The conditioned medium was the extract of the dressing in the normal cell medium for 24 h and the proportion of substance in the extract was shown in the Table S3. We suspected that slight substances of the dressing contain dBAM and chitosan were transferred into the medium during the process of preparation of conditioned medium. Hence, the cell viabilities of dressing groups were above 100% due to the substances of dBAM and chitosan.

### 3.5. Evaluation of the Wound Healing Ability

**Figure 6:**
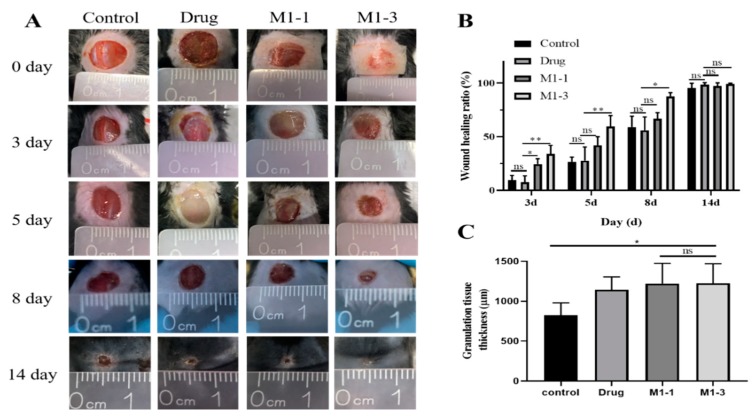
(**A**) the healing processes of wounds treated of diabetic mice, (**B**) wound healing ratios analysis, (**C**) granulation tissue thickness of the wounds at 14 day (ns: *p* > 0.05; *: *p* < 0.05; **: *p* < 0.01).

In vivo healing experiment was used to evaluate the wound healing effectiveness of M1-1 and M1-3 (Figure 6A). During the complex processes of wound healing, the wound area and the skin thickness were detected to evaluate the degree of wound healing. At the beginning, the experimental mice were depilated the back hair and operated with a full-thickness skin wound with approximately 10 mm diameter. They were divided into four groups (the 3M membrane group as control, the drug group as positive control, the M1-1 group, and the M1-3 group) randomly. M1-3 group presents better wound healing at each period obviously, especially at days 3, 5, and 8 (Figure 6B). The wound healing ratios of M1-3 were 34% at day 3, 59.5% at day 5, and 87.67% at day 8 on average. In addition, the granulation tissue thickness was tested to reflect the wound healing process. At day 14, the wound treated with BAMCSM (M1-1, M1-3) formed the thickest granulation tissue exhibiting 1225 μm in average which was better than other groups, even 400 μm thicker than control group (Figure 6C).

### 3.6. Histological Evaluation of Wound Healing

**Figure 7:**
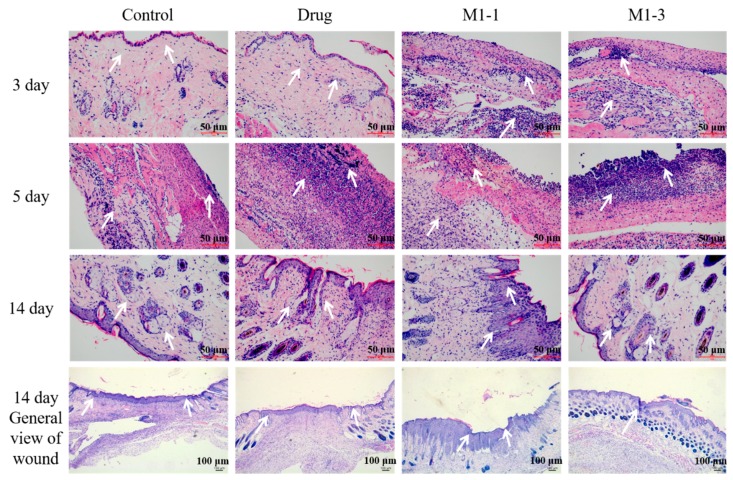
H&E staining of skin tissues at days 3, 5, and 14.

To observe the wound healing process of the angiogenesis and tissue regeneration, H&E staining method was adopted. As the skin repair is a complicate process which can be divided into three stages of tissue inflammation, formation and healing. In the early stage of wound healing, inflammatory cells are the most important and can produce many cell factors such as pro-inflammatory cytokines to initial the wound inflammation. As shown in Figure 7, at day 3, group M1-1 and M1-3 showed a more severe inflammation which indicated that the M1-1 and M1-3 could stimulate the tissue into the first stage of repair process earlier than others. At day 5, the healing process of M1-1 and M1-3 group was markedly accelerated, demonstrated by an elevated number of fibroblast infiltration and neovascularization compared to control and drug groups. It can be speculated that this process started the tissue formation stage of wound healing. In contrast, negative and drug groups showed more serious inflammation which was the typical performance of tissue inflammation stage. At day 14, the M1-3 group showed a thicker and better healing, where the sebaceous gland and hair follicles were observed to be arranged in order, whereas control and drug groups demonstrated inferior healing. Additionally, the healing condition of M1-1 group was second only to M1-3 group.

**Figure 8:**
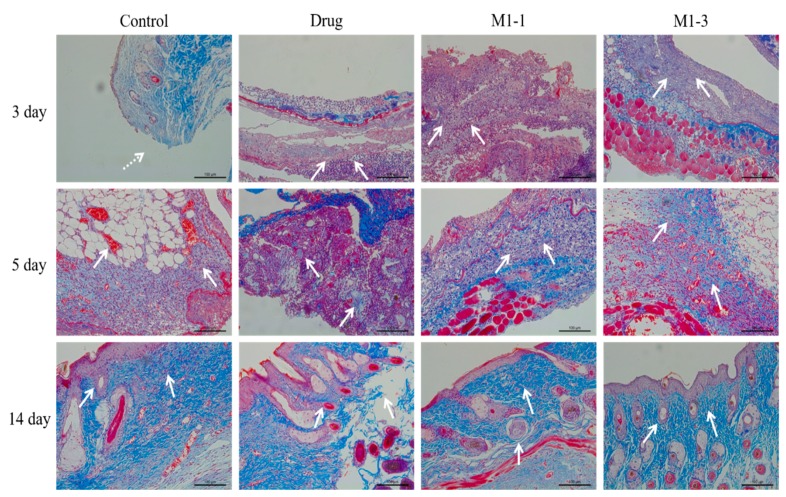
Masson staining of skin tissues at days 3, 5, and 14.

Massonstaining of different groups was performed at day 3, 5 and 14 to observe the collagen generation within the regenerated tissue during the repair process (Figure 8). At day 3, almost no collagen fiber was formed in each group. At day 5, M1-1 and M1-3 group produced collagen more than the control and drug groups, where a low level of collagen was visualized. After day 14, the M1-3 group displayed matured bundled collagen fibers with an organized parallel fibrous structure, whereas the tissue of control and drug groups showed partial and spare collagen generation.

## 4. Discussion

In this paper, dBAM and CS has been used to generate a membrane which was subsequently applied for the treatment of diabetic wound healing. The double-layer wound dressing (M1-3) exhibited as the best group for wound healing. According to the results, we can conclude that the BAMCSM has good biocompatibility, high elasticity, air permeability, and can promote the wound healing. The analysis is mainly attributed to the following points: firstly, the properties of dBAM and CS are very suitable as wound dressing; secondly, the dense layer of dBAM can prevent bacterial penetration and dehydration of wound surface, while allowing the drainage of wound and support a great microenvironment to accelerate the wound healing due to the rich collagen and growth factors [40], at last, the porous structure of sponge-like chitosan was in favor of wound microenvironment. It not only remains the advantage such as great absorption capacity for fluid, fills the deep wound and inhabits the bacterial growth [41,42], but also overcomes the main disadvantage such as poor mechanical property which is unfavorable for the debridement and wound healing [43].

Since the chronic wound of diabetes is a multifactorial complication that result particularly as a consequence of peripheral neuropathy, impaired vascular function, impaired angiogenesis, and/or chronic inflammation, in the future, we can consider using the combination of BAMCSM and anti-inflammatory drugs to improve the therapeutic effect. The result of animal studies indicated that the thickness of chitosan layer was vital to wound healing. However, the relationship between the thickness of membrane and the therapeutic effects needs further discussion in the future studies.

## 5. Conclusions

In summary, we developed a double-layer membrane using chitosan and dBAM with excellent biocompatibility through simple synthetic procedures. The swelling and mechanical abilities of BAMCSM were improved apparently compared to the single sponge-like chitosan membrane and dBAM. The chitosan layer was demonstrated to increase the hemostatic ability of wound dressing. Furthermore, we evaluated the curative ability of BAMCSM in diabetic wound models. The results indicated that the M1-3 was able to support the best microenvironment in favor of wound healing. The double-layer membrane was concluded to promote the wound into the stage of tissue inflammation earlier and finally showed a thicker and better healing effect, where the sebaceous gland, hair follicles and collagen fiber with organized parallel structure were observed. The results showed the double-layer membrane could be a great candidate for wound dressing applications.

## 6. Patents

We applied a PCT (Patent Cooperation Treaty, Application number PCT/CN2019/122412) earlier.

## Supplementary Materials

The following are available online at https://www.mdpi.com/2073-4360/12/3/535/s1, Figure S1: DAPI staining of BAM with different conditions, Figure S2: ART-FTIR spectra of chitosan, PEGDGE, and M1-1, Figure S3: swelling ratios of dBAM, M1-1, and M1-3 (0–6 h), Table S1: the components of decellularized bovine amniotic membrane, Table S2: the theoretical dry weight of each membranes (rectangular shape of 10 cm long and 3 cm wide), Table S3: the proportion of substances in the extract.
