# Supplementary material for: A Sponge-Like Double-Layer Wound Dressing with Chitosan and Decellularized Bovine Amniotic Membrane for Promoting Diabetic Wound Healing
Source: Polymers (Basel). 2020 Mar 2;12(3):535. doi: 10.3390/polym12030535 (PMC7182886; doi:10.3390/polym12030535)
Supplement: Supplementary file 1 [file polymers-12-00535-s001.pdf]

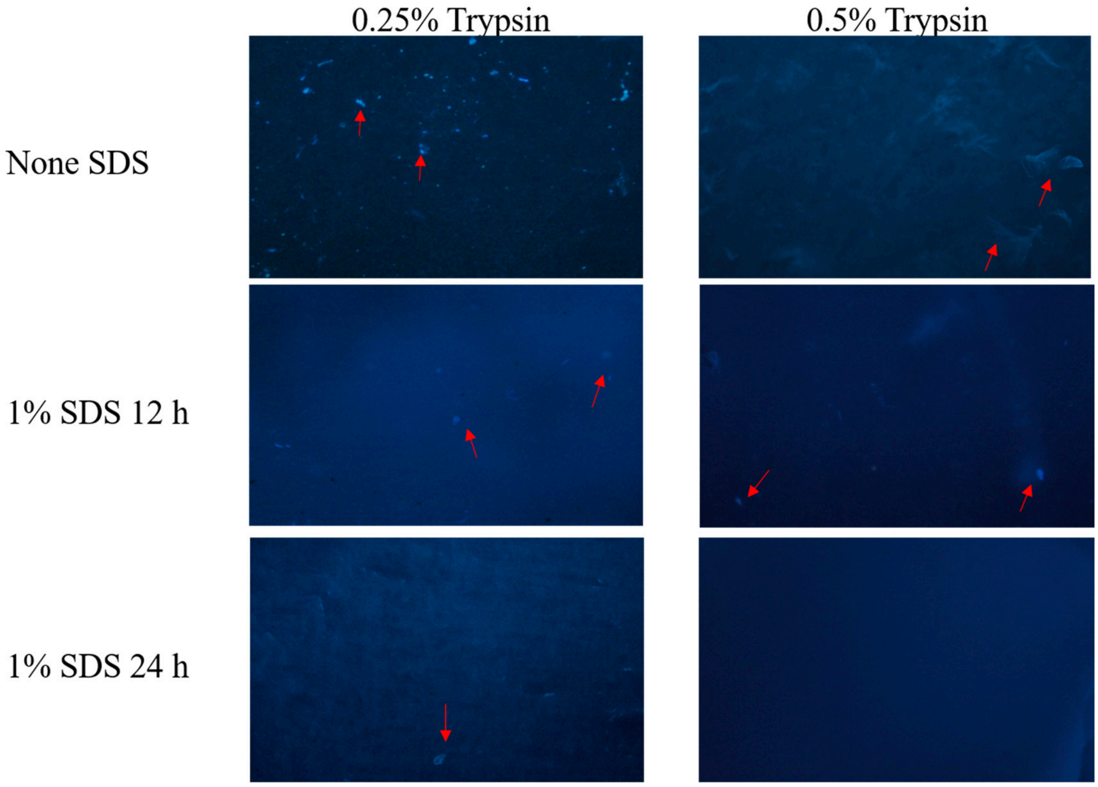

Figure S1. DAPI staining of BAM with different conditions.

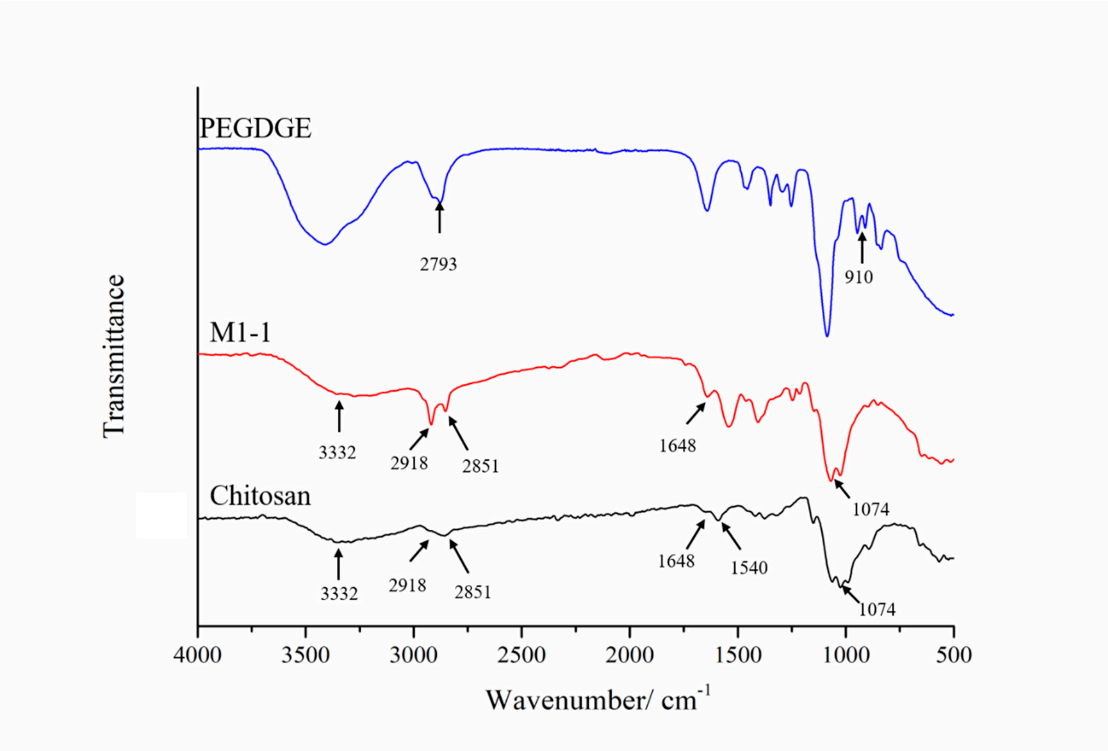

Figure S2. ART-FTIR spectra of chitosan, PEGDGE and M1-1.

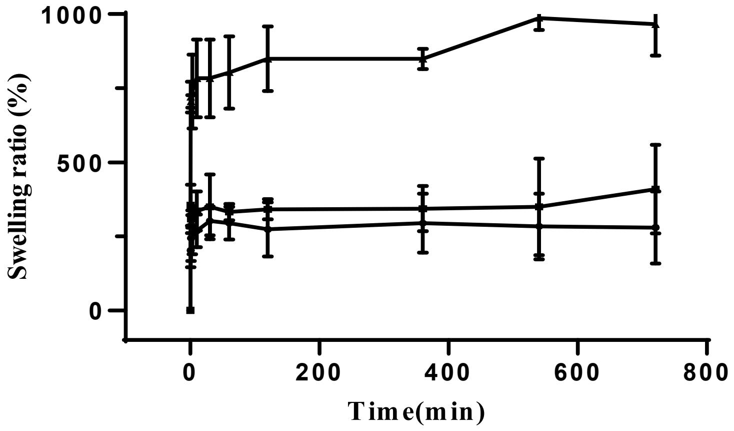

Figure S3. Swelling ratios of dBAM, M1-1 and M1-3 (0-6 h).

Table S1 The components of decellularized bovine amniotic membrane.

Component	Detection method	Content (%)
Water	Freeze-drying	11.18
Total protein (including collagen)	Kjeldahl determination	66.62
Collagen	Hydroxyproline method	21.77
Polysaccharide	The method of anthrone	22.25
Fat	Soxhlet extraction	0.12

Table S2 The theoretical dry weight of each membranes (rectangular shape of 10 cm long and 3 cm wide).

Membranes	dBAM	M1-1	M1-3
dry weight	10 mg	20 mg	40 mg

Table S3. The proportion of substances in the extract.

Condition mediums of different dressings	The proportion of substances in the extract		
	cell medium	dBAM	chistosan
**dBAM (6 cm²/mL)**	1 ml	2 mg	0 mg
**M1-1 (6 cm²/mL)**	1 ml	2 mg	2 mg
**M1-3 (3 cm²/mL)**	1 ml	1 mg	3 mg
